# Successful reconstruction of large oropharyngeal defect with pectoralis major myocutaneous flap in a four-year-old boy with recurrent fibromatosis

**DOI:** 10.1186/1477-7819-5-11

**Published:** 2007-01-29

**Authors:** Sajid S Qureshi, Quazi G Ahmed, Prabha S Yadav

**Affiliations:** 1 Department of Paediatric Surgical Oncology, Tata Memorial Hospital. Ernest Borges Road, Parel, Mumbai 400012, India; 2 Department of Plastic & Reconstructive Services, Tata Memorial Hospital. Ernest Borges Road, Parel, Mumbai 400012, India

## Abstract

**Background:**

Pectoralis major myocutaneous (PMMC) flap continues to be the workhorse in head and neck reconstruction. Although free tissue transfer has revolutionized the reconstruction in cancers of the oral region, PMMC is still considered a readily accessible source of vascularized soft tissue available to the reconstructive surgeon and especially in most developing nations where due to the cost, time, expertise, or infrastructural constraints free flaps cannot be generally offered. Although commonly used in adults, it has been hardly described for reconstruction in children.

**Case presentation:**

We present a 4-year-old child with recurrent fibromatosis of the oropharyngeal region where the PMMC was used for reconstruction of the surgical defect and to the best of our knowledge is the youngest patient undergoing reconstruction with PMMC for neoplastic lesion of the head and neck.

**Conclusion:**

The PMMC flap is justifiably a popular flap that continues to command an important place in the head and neck surgeon's reconstructive armamentarium.

## Background

Initially described by Ariyan in 1979, the PMMC flap has been used to reconstruct oncologic head and neck defects after either primary extirpation or surgical salvage following radiation failure [1,2]. The versatility of the PMMC is evident from its very wide range of use. The flap can be used for defects in the head and neck area including the oral cavity, neck, and maxilla as well as temporo-orbital area [3]. A tube PMMC can also be used to reconstruct the pharynx and the cervical oesophagus. Extending its versatility we present the use of PMMC for mucosal and soft tissue cover in a 4-year-old child.

## Case presentation

**Figure 1:**
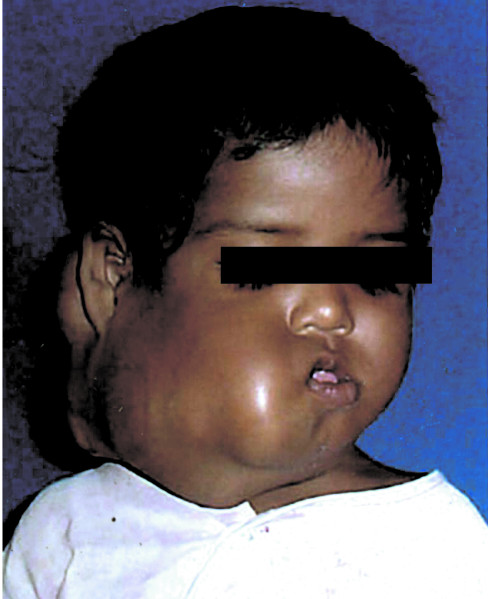
Clinical photograph of the patient at presentation.

**Figure 2:**
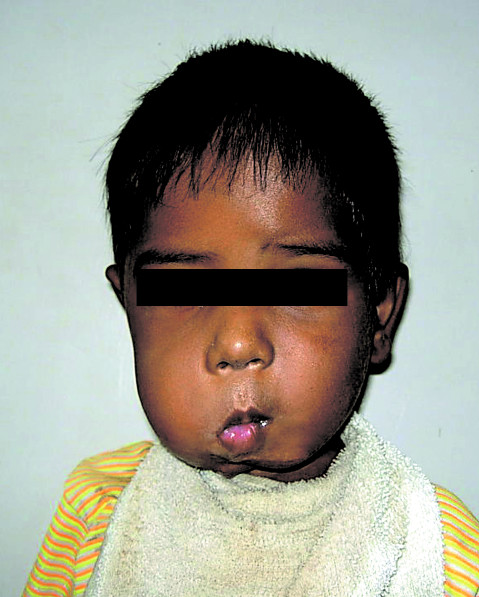
Clinical photograph of the patient after recurrence.

**Figure 3:**
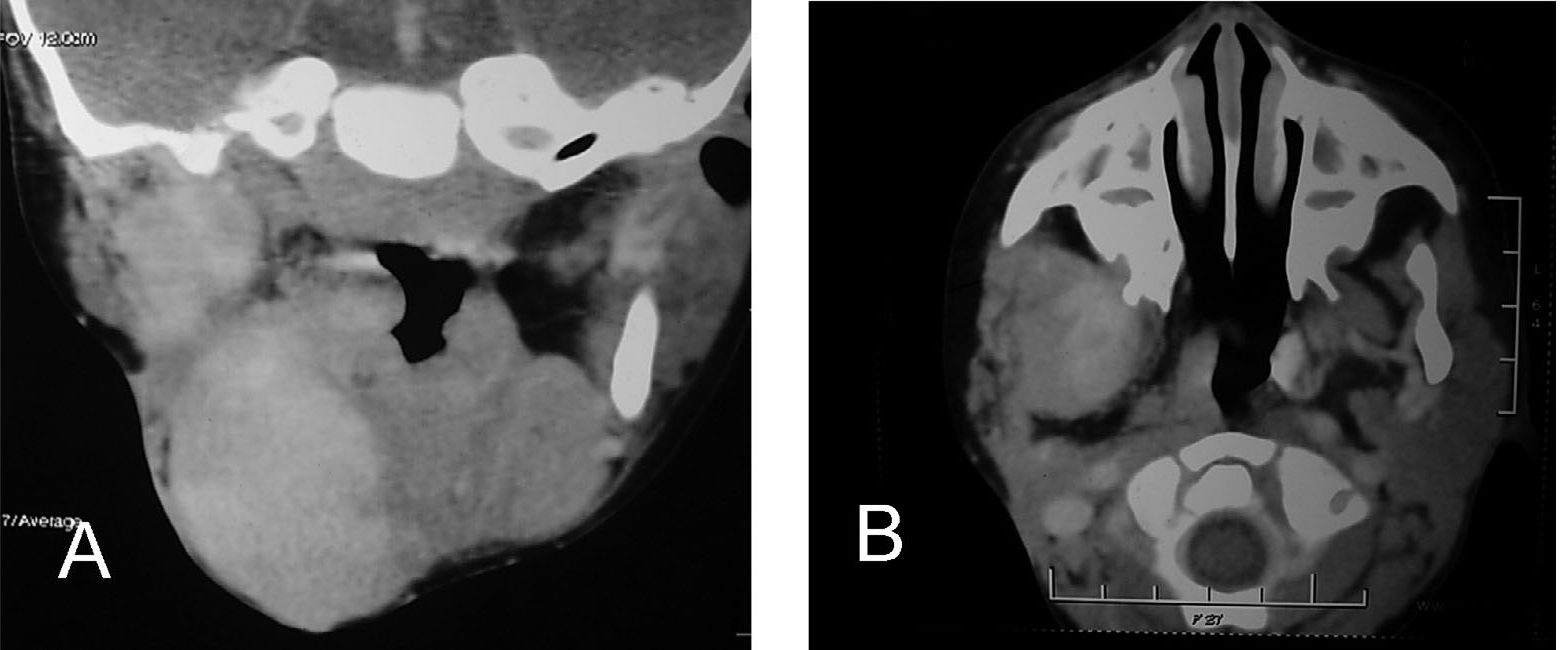
A CT scan revealing recurrence at the floor of mouth and presence of tumor in the infratemporal fossa and base of skull.

**Figure 4:**
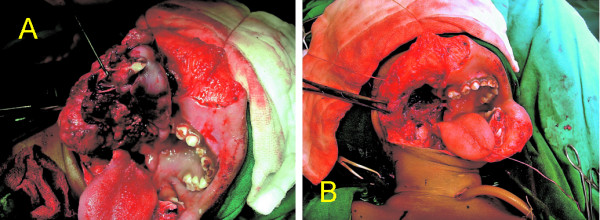
A&B). Intraoperative photograph revealing the tumor being resected and the surgical defect after excision.

**Figure 5:**
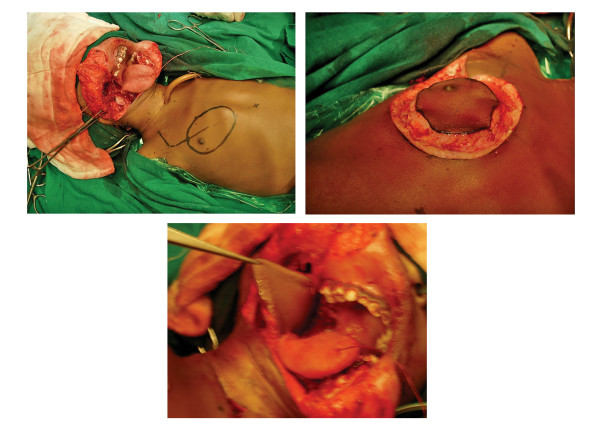
A, B & C). Intraoperative photograph showing the skin paddle of PMMC and its suturing to the surgical defect.

A 3-year-old boy presented with a large bosselated swelling over the cheek extending to the submandibular, submental, postauricular region and which was still further increasing in size (Figure 1). A trucut^® ^biopsy was suggestive of fibromatosis. Computerized tomogram (CT) scan revealed a large soft tissue mass infiltrating the massetter, parotid gland, floor mouth, submandibular gland, the lateral oro-nasopharyngeal wall and the pterygoid muscle. The horizontal ramus of mandible was eroded and the mass extended across the midline. Excision of the mass with segmental mandibulectomy was performed. Reconstruction with a free fibula was deferred for a later stage in view of the young age, large mass and concern of recurrent disease. Since the mucosal defect following excision was not large enough primary closure was performed. Histopathology confirmed juvenile fibromatosis and all cut margins of resection were free of tumour. The patient presented after 13 months with recurrent swelling over the cheek with significant difficulty in swallowing and constant dribbling of saliva due to inadequate mouth closure (Figure 2). A CT scan revealed presence of a large recurrent mass in the floor of mouth, infiltrating the infratemporal fossa (ITF) and reaching up to the base of skull (Figure 3). In view of the extensive recurrence low dose chemotherapy (weekly vinblastine, methotrexate and tamoxifen) was started. After completion of 12 weeks of chemotherapy, a partial response was achieved. Excision of the mass with ITF clearance was performed subsequently. A portion of tongue and its base along with the lateral oropharyngeal and the soft palate was also excised (Figure. 4) The defect following resection was large and a free flap was not feasible as the external carotid artery was ligated at previous surgery. Reconstruction with a PMMC flap for providing mucosal cover and soft tissue bulk for defect was considered. In view of the large mucosal defect a large skin paddle was taken incorporating the nipple-areola complex. The technique for harvesting the PMMC has been described previously [4] (Figure 5). The edges of the flap were sutured circumferentially to the mucosa of the oral cavity except superiorly where the mucosa was deficient; the flap was anchored to the hard palate with non-absorbable sutures.

Patient had an uneventful recovery and was discharged after ten days. The flap was well taken without any necrosis or other complication. Guide bite prosthesis was used after a week of surgery to prevent malocclusion. Histopathological examination revealed all cut margins to be free of tumour. Patient further received 12 weeks of low dose chemotherapy. At one year follow-up the patient is having normal speech and swallowing and is free from recurrent disease.

## Discussion

PMMC flap is a versatile and the most commonly used flap for head and neck reconstruction. Even with the worldwide use of free flaps, they are still the mainstay reconstructive procedures in many centres. In the largest reported series of 500 patients, Milenovic and colleagues reported an overall complication rate of 33%, with only 2% of cases involving total flap necrosis [5]. They concluded that the PMMC flap continues to be the most universal major flap in head and neck reconstruction. Similar observations were made by Vartanian and colleagues in their series of 437 patients [6]. They also observed that the PMMC flap remains an important reconstructive method, and can be done with low risk and acceptable morbidity. Liu and colleagues used PMMC for reconstruction of oral cavity, pharynx, and neck or face as primary reconstructive procedures, or salvage procedures for reconstruction after fistula, flap failure, osteoradionecrosis, and internal jugular vein rupture [3]. In the present case although a free flap reconstruction using a transverse rectus abdominis flap with vascular anastomosis in the opposite neck was deliberated at the second surgery it was considered too extensive for a 4-year-old child hence was deferred. The patient had an excellent recovery with an acceptable cosmesis and function (swallowing, speech, tongue control and oral competence) and is free of disease at one-year follow-up. Although free flaps should be ideally considered in all situations, PMMC can be considered as a reserve in difficult situations and where infrastructural support constrains the liberal use of free flaps.

## Conclusion

The PMMC flap is justifiably a popular flap that continues to command an important place in the head and neck surgeon's reconstructive armamentarium.

## Competing interests

The author(s) declare that they have no competing interests.

## Authors' contributions

**QSS **– lead pediatric oncosurgeon involved in this patient's management, research, acquisition of data, acquisition of consent, writing, drafting and critical review and revision of manuscript.

**QAG **– involved in discussions leading to manuscript preparation and critical review of manuscript

**PYS **– contributed to manuscript conception, organizing manuscript, critical reviews of manuscript.

All authors have read and approved the final manuscript

## References

[B1] Ariyan S (1979). The pectoralis major myocutaneous flap. Plast Reconstr Surg.

[B2] Ariyan S (1979). Further experiences with the pectoralis major myocutaneous flap for the immediate repair of defects from excisions of head and neck cancers. Plast Reconstr Surg.

[B3] Liu R, Gullane P, Brown D, Irish J (2001). Pectoralis major myocutaneous pedicled flap in head and neck reconstruction: retrospective review of indications and results in 244 consecutive cases at the Toronto General Hospital. J Otolaryngol.

[B4] Qureshi SS, Chaukar DA, Dcruz AK (2004). A simple technique of raising the pectoralis major myocutaneous flap along the deltopectoral groove. J Surg Oncol.

[B5] Milenovic A, Virag M, Uglesic V, Aljinovic-Ratkovic N (2006). The pectoralis major flap in head and neck reconstruction: first 500 patients. J Craniomaxillofac Surg.

[B6] Vartanian JG, Carvalho AL, Carvalho SM, Mizobe L, Magrin J, Kowalski LP (2004). Pectoralis major and other myofascial/myocutaneous flaps in head and neck cancer reconstruction: Experience with 437 cases at a single institution. Head Neck.

